# Intra-Tumoral Delivery of IL-27 Using Adeno-Associated Virus Stimulates Anti-tumor Immunity and Enhances the Efficacy of Immunotherapy

**DOI:** 10.3389/fcell.2020.00210

**Published:** 2020-03-27

**Authors:** Aiyan Hu, Miao Ding, Jianmin Zhu, Jin-Qing Liu, Xueliang Pan, Kalpana Ghoshal, Xue-Feng Bai

**Affiliations:** ^1^Institute of Pediatric Translational Medicine, Shanghai Children’s Medical Center, Shanghai Jiao Tong University School of Medicine, Shanghai, China; ^2^Department of Pathology, The Ohio State University Wexner Medical Center, Columbus, OH, United States; ^3^The Comprehensive Cancer Center, The Ohio State University Wexner Medical Center, Columbus, OH, United States; ^4^Center for Biostatistics, The Ohio State University, Columbus, OH, United States

**Keywords:** IL-27, recombinant adeno-associated virus, B16 melanoma, J558 plasmacytoma, Tregs, PD-1 blockade therapy, T cell adoptive transfer

## Abstract

IL-27 is an anti-inflammatory cytokine that has been shown to have potent anti-tumor activity. We recently reported that systemic delivery of IL-27 using recombinant adeno-associated virus (rAAV) induced depletion of Tregs and significantly enhanced the efficacy of cancer immunotherapy in a variety of mouse tumor models. A potential caveat of systemic delivery of IL-27 using rAAV is that there is no practical method to terminate IL-27 production when its biological activity is no longer needed. Therefore, in this work, we tested if directly injecting AAV-IL-27 into tumors could lead to similar anti-tumor effect yet avoiding uncontrolled IL-27 production. We found that high levels of IL-27 was produced in tumors and released to peripheral blood after AAV-IL-27 intra-tumoral injection. AAV-IL-27 local therapy showed potent anti-tumor activity in mice bearing plasmacytoma J558 tumors and modest anti-tumor activity in mice bearing B16.F10 tumors. Intra-tumoral injection of AAV-IL-27 induced infiltration of immune effectors including CD8^+^ T cells and NK cells into tumors, caused systemic reduction of Tregs and stimulated protective immunity. Mechanistically, we found that IL-27 induced T cell expression of CXCR3 in an IL-27R-dependent manner. Additionally, we found that AAV-IL-27 local therapy had significant synergy with anti-PD-1 or T cell adoptive transfer therapy. Importantly, in mice whose tumors were completely rejected, IL-27 serum levels were significantly reduced or diminished. Thus, intra-tumoral injection of AAV-IL-27 is a feasible approach that can be used alone and in combination with anti-PD-1 antibody or T cell adoptive transfer for the treatment of cancer.

## Introduction

Cancer immunotherapies based on blockade of immune checkpoints (Phan et al., 2003; Topalian et al., 2012; Hamid et al., 2013) have achieved significant success. However, a majority of patients with advanced cancer are not sensitive to this type of immunotherapy. Although factors responsible for cancer resistance to immunotherapy are not fully understood, the following factors are considered important. *First*, lack of pre-existing T cell infiltration in the tumor microenvironment (TME) is considered to be the most important factor for anti-PD-1 resistance (Tumeh et al., 2014). *Second*, although not well-established in human cancer, regulatory T cells (Tregs) in the TME contribute to anti-PD-1 resistance in mouse models (Ngiow et al., 2015). Tregs expand in cancer patients and are enriched in cancer lesions (Curiel et al., 2004). *Third*, although not absolute, tumor expression of PD-L1 is another potentially important factor. In cancer types such as non-small cell lung carcinoma, bladder cancer and melanoma, PD-L1 immunohistochemistry has identified patients with a higher likelihood of treatment response (Topalian, 2017). Thus, developing novel strategies that can overcome these limitations is critical to enhancing the efficacy of current cancer immunotherapies.

Although the role of IL-27 in tumor immunity has been appreciated for more than a decade, developing IL-27 into a therapeutic to enhance tumor immunity has not been well achieved. Recombinant adeno-associated viral vectors (rAAV) are highly versatile gene delivery agents for gene therapy. The lack of immunogenicity and toxicity make rAAV the vector of choice for human clinical trials (Aalbers et al., 2011). Recently (Zhu et al., 2018), we have produced IL-27-expressing rAAV (AAV-IL-27) that can efficiently produce IL-27 in recipient mice and made the following novel observations. First, AAV-IL-27 significantly inhibits the growth of a broad-spectrum of tumor types in mice. Second, AAV-IL-27 treatment results in dramatic reduction of Tregs without causing autoimmunity. Third, AAV-IL-27 therapy shows strong synergy with PD-1 antibody in inhibiting tumor growth.

A potential caveat of systemic delivery of IL-27 using rAAV is that there is no practical method to terminate IL-27 production when its biological activity is no longer needed. In this study, we tested the approach of directly injecting AAV-IL-27 into tumors and determined its anti-tumor efficacy. We found that intra-tumoral administration of AAV–IL-27 induces infiltration of immune effectors including CD8^+^ T cells and NK cells into tumors, causes systemic reduction of Tregs and stimulates protective immunity. Mechanistically, we found that AAV-IL-27 induces T cell expression of CXCR3 in an IL-27R-dependent manner. Additionally, we found that AAV-IL-27 local therapy has significant synergy with anti-PD-1 or T cell adoptive transfer therapy. Importantly, in mice whose tumors were completely rejected, IL-27 serum levels were significantly reduced or diminished.

## Materials and Methods

### Mice and Tumor Cells

C57BL/6, BALB/c, and IL27R^–/–^ mice were purchased from The Jackson Laboratory and were maintained in the animal facilities of the Ohio State University. Stat1^–/–^ BALB/c mice were described before (Zhu et al., 2018). B16.F10 melanoma cells and plasmacytoma J558 cells were originally obtained from ATCC and used after a few passages *in vitro*. These cancer cells were maintained in RPMI1640 medium (Gibco) supplemented with 100 μg/ml penicillin, 100 μg/ml streptomycin, and 10% FBS (Gibco).

### Treatment of Mice With Tumors Using AAV

Production of rAAV-IL-27 has been described previously (Zhu et al., 2018). To establish tumors in mice, indicated numbers of cancer cells were injected into each C57BL6 or BALB/c mouse s.c. in 100 μl of PBS. AAV-IL-27 or AAV-ctrl viruses were diluted in PBS containing the indicated quantity of AAV virus, and were injected into the established tumors in a total volume of 50 μl. The length and width of tumors were measured using a digital caliper every 2 or 3 days. The tumor volume was calculated according to the formula volume (V) = ab^2^/2, where a represents length and b represents width. In some experiments, starting on the day of AAV treatment, mice were also treated with 250 μg/mouse of anti-PD-1 (RMP1-14) or an isotype-matched control antibody (anti-rat IgG2a; 2A3) i.p. at 3-day intervals for up to 4 times. Anti-PD-1 antibody and isotype-matched control antibody were purchased from BioXcell.

### Flow Cytometry

FITC-, PE-, PE-CY7, APC-, APC-CY7 or Percp-labeled antibodies to CD3 (145-2C11), CD45 (30-F11), CD4 (GK1.5), CD8α (53-6.7), CD11b (M1/70), CD11c (N418), B220 (RA36B2), CD25 (PC61), NK1.1 (PK136), PD-L1 (MIH5), FoxP3 (NRRF-30), IFN-γ (XMG1.2), TNF-α (MAb11), CD49 (DX5), and CXCR3 (CXCR3-173) and isotype-matched control antibodies were purchased from Biolegend (antibodies to CD3, CD4, CD8, CD45,CD11b, CD11c, B220, CD25, PD-L1, FoxP3, IFN-γ, TNF-α, CD49, CXCR3, and control antibodies) or BD Biosciences (antibodies to NK1.1 and control antibodies). For identification of cellular phenotypes, disassociated cells from tumors or spleens were suspended in PBS containing 1% bovine serum albumin and incubated with the antibodies on ice for 30 min. Cells were fixed in 1% paraformaldehyde in PBS after washing. For intracellular cytokine staining, cells were stimulated in culture medium for 4 h in the presence of Leukocyte Activation Cocktail, with BD Golgi^*Stop*^ (1: 500; BD Biosciences). Viable cells were then fixed and permeabilized with transcription staining buffer set (eBioscience) and stained with respective antibodies to cytokines. FoxP3 staining was performed according to manufacturer’s protocol (eBioscience). Stained cells were analyzed on a FACSCalibur or FACS Canton flow cytometer, and data were analyzed using the flowjo software.

### ELISA

ELISA kit for the detection of IL-27 was purchased from eBiosciences. Standard procedures were followed to detect releases of cytokines in supernatants of cultured cells in a variety of settings or blood of AAV-treated mice.

### Real Time RT-PCR

Quantitative real-time PCR was performed using previously determined conditions (Liu et al., 2013). The relative amount of mRNA was calculated by plotting the Ct (cycle number) and the average relative expression for each group was determined using the comparative method (2^–Δ^ ^Δ^ ^*Ct*^). The following primers were used for amplifying IL-27 and β-actin genes: IL-27: 5′-TCTGAGGTTCAGGGCTATGT-3′(forward) and 5′- TCAGGGAAACATTGGGAAGATG-3′(reverse); β-actin: 5′- GAGACCTTCAACACCCCAGC-3′ (forward) and 5′- ATGTCACGCACGATTTCCC-3′ (reverse).

### Cytotoxicity Assay

A flow cytometry-based cytotoxicity assay was used to measure *in vitro* cellular cytotoxicity of tumor-infiltrating lymphocytes (TILs) to J558 target cells as previously described (Liu et al., 2013).

### Generation of Tumor-Infiltrating Lymphocytes and Adoptive Transfer

Tumor infiltrating CD8^+^ T cells were isolated from established J558 tumors. Briefly, 5 × 10^6^ J558 cells diluted in PBS were injected into BALB/c mice s.c. in a volume of 100 ul. When tumors reached 1 cm in length, mice were sacrificed and mouse tumors were dissected, ground to single cell suspensions. CD8^+^ TILs were isolated by using CD8 MicroBeads (MACS) and cultured in RPMI 1640 (Gibico) with 10% fetal calf serum, 2-mercaptoethanol(invitrogen), HEPES(invitrogen), penicillin/streptomycin (invitrogen), recombinant human IL-2 (50IU/ml, Peprotech), and 20 ng/ml anti-CD3 antibody (2C11, Biolegend) in the presence of 2 × 10^6^/ml irradiated syngeneic splenocytes (2000 rad). TIL cultures were split when confluent and reseeded at 2–3 × 10^5^/ml in culture medium. To treat mice with established J558 tumors, 5 × 10^6^ *in vitro*-cultured TIL CD8^+^ cells were injected into each tumor-bearing mice i.v.

#### Statistics

One way ANOVA, student’s *t* test and log-rank tests were used for statistical analyses. The GraphPad Prism software was used for all the analyses.

## Results

### Intra-Tumoral Administration of AAV-IL-27 Inhibits Tumor Growth and Stimulates Anti-tumor Immunity

**FIGURE 1:**
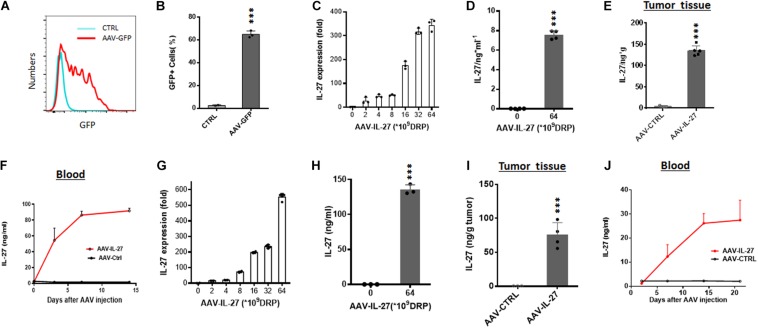
AAV can efficiently infect tumor cells *in vitro* and *in vivo*. **(A,B)** B16.F10 melanoma cells were incubated with AAV-GFP at a concentration of 5 × 10^9^ DRP (DNase resistant particles) for 48 h and GFP-positive melanoma cells were analyzed and quantified by flow cytometry. **(C,D)** B16.F10 melanoma cells were infected with AAV-IL-27 at various concentrations, and the expression of IL-27 mRNA **(C)** in melanoma cells and IL-27 protein **(D)** in the cell culture supernatants were quantified by qPCR or ELISA. **(E,F)** AAV-IL-27 was directly injected into established B16 melanoma tumors in C57BL/6 mice and the blood samples were collected at different time points. Mice were sacrificed 2 weeks after AAV injection and the concentrations of IL-27 in tumor tissues **(E)** and blood **(F)** were quantified by ELISA. **(G,H)** J558 plasmacytoma cells were infected with AAV-IL-27 at various concentrations, and the expression of IL-27 mRNA **(G)** in J558 cells and IL-27 protein **(H)** in the cell culture supernatants were quantified by qPCR or ELISA. **(I–J)** AAV-IL-27 was directly injected into established J558 tumors in BALB/c mice and the blood samples were collected at different time points. Mice were sacrificed 3 weeks after AAV injection and the concentrations of IL-27 in tumor tissues **(I)** and blood **(J)** were quantified by ELISA. Data are expressed as Mean ± SD of 3-5 samples in each group/per time point. *P < 0.05, **P < 0.01, ***P < 0.001 by one-way ANOVA or student’s *t* test.

To determine if AAV is a suitable vector for tumor local delivery of therapeutics, we generated rAAV vectors that express GFP (AAV–GFP) and tested if AAV-GFP could infect B16 melanoma cells. After incubation with AAV-GFP for 48 h, more than 60% B16 melanoma cells expressed GFP (Figures 1A,B). Moreover, injection of AAV-GFP into established B16 tumors resulted in the GFP expression at the injection sites (Supplementary Figure S1). Thus, it appears that B16 melanoma cells are sensitive to AAV infection *in vitro* and *in vivo*. Next, we tested if IL-27 expressing rAAV (AAV-IL-27) infection of B16 cells could lead to IL-27 production. As shown in Figure 1C, AAV-IL-27 infected B16 cells dose-dependently, as quantified for IL-27 mRNA by qPCR. AAV-IL-27 infection of B16 cells also resulted in release of IL-27 protein in the culture supernatant, as determined by ELISA after 48 h incubation (Figure 1D). To determine if AAV-IL-27 intra-tumoral delivery leads to IL-27 production *in vivo*, we directly injected AAV-IL-27 into established B16 tumors in C57BL/6 mice and examined IL-27 in blood samples at different times after AAV-IL-27 injection. As shown in Figure 1E, AAV–IL-27 intra-tumor administration resulted in IL-27 production in tumors. We could also detect sustained IL-27 production in blood after AAV-IL-27 intra-tumoral injection (Figure 1F). Similarly, we found that AAV-IL-27 could also infect mouse plasmacytoma J558 cells *in vitro* (Figure 1G), which resulted in IL-27 protein production during *in vitro* culture (Figure 1H). Injection of AAV-IL-27 into established J558 tumors in mice also resulted in IL-27 production in tumors (Figure 1I) and blood (Figure 1J). Thus, intra-tumoral injection of AAV-IL-27 resulted in IL-27 production in tumors, which was subsequently released to blood.

**FIGURE 2:**
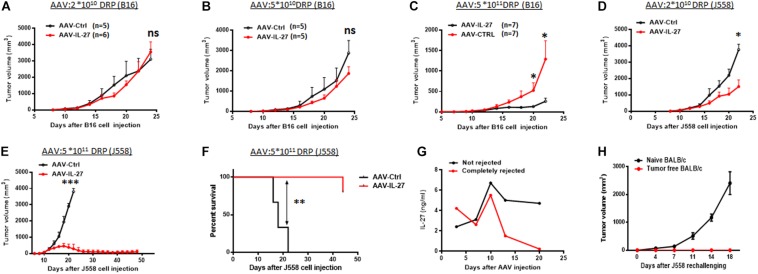
Intra-tumoral injection of AAV-IL-27 inhibits tumor growth and stimulates protective immunity in mice. **(A–C)** B16.F10 cells (2 × 10^5^ cells) were injected into C57BL/6 mice s.c. 7 days later, AAV-IL-27 or AAV-ctrl virus were directly injected into the established tumors at the indicated dose. **(D–F)** J558 cells (5 × 10^6^ cells) were injected into BALB/c mice s.c. Five days later, mice were treated with AAV-IL-27 or AAV-ctrl virus intra-tumorally at the indicated doses. Tumor volume changes **(A–E)** and mice survival **(F)** were recorded. **(G)** Serum IL-27 concentrations in mice with J558 tumors or mice whose tumors were rejected. **(H)** J558 cells were injected into mice whose tumors were rejected after AAV-IL-27 therapy or naïve BALB/c mice, and tumor growth was compared. Data shown represent 2–3 experiments with similar results. ns: no significant difference. *P < 0.05, **P < 0.01, ***P < 0.001by One-way ANOVA or Log-rank test.

To test if intra-tumoral delivery of AAV-IL-27 inhibits tumor growth, we injected AAV–IL-27 into B16 melanoma tumors in mice at various doses. At lower doses, AAV-IL-27 did not inhibit B16 tumor growth significantly (Figures 2A,B), while at a higher doses (Figure 2C), intra-tumoral injection of AAV-IL-27 significantly inhibited the growth of B16 tumors. Similarly, at a lower dose, injection of AAV-IL-27 into established J558 tumors resulted in a slight inhibition of tumor growth (Figure 2D), while at a high dose, mice had nearly complete tumor rejection (Figure 2E) with long term tumor free survival (Figure 2F). We examined blood IL-27 levels in two typical mice whose tumors were not rejected or completely rejected, and found that tumor rejection also resulted in IL-27 reduction (Figure 2G). Moreover, we found mice that rejected J558 tumors were completely resistant to J558 tumor cell re-challenging (Figure 2H). Thus, AAV–IL-27 intra-tumoral delivery is not only an effective treatment in experimental mouse tumor models, it also induces protective immunity.

### Intra-Tumoral Administration of AAV-IL-27 Enhances Accumulation of T and NK Cells in Tumors and Causes Systemic Reduction of Tregs

**FIGURE 3:**
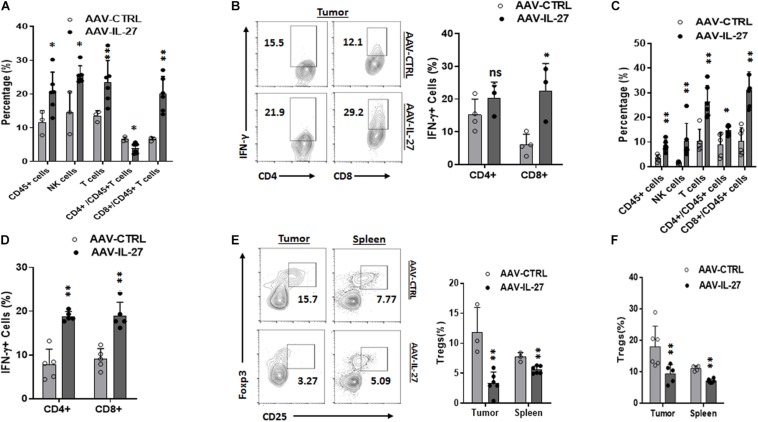
Intra-tumoral injection of AAV–IL-27 alters tumor immune microenvironment. **(A,B)** B16.F10 cells (2 × 10^5^) were injected into C57BL/6 mice s.c. Seven days later, mice were treated with AAV-IL-27 or AAV-ctrl virus intra-tumorally at a dose of 2 × 10^11^ DRP/mouse. Mice were sacrificed on day 21, and populations of tumor infiltrating lymphocytes **(A)** and IFN-γ production by tumor infiltrating T cells **(B)** were analyzed by flow cytometry. **(C,D)** J558 cells (5 × 10^6^) were injected into BALB/c mice s.c. Seven days later, mice were treated with AAV-IL-27 or AAV-ctrl virus intra-tumorally at a dose of 5 × 10^10^ DRP/mouse, and mice were sacrificed on day 21, and populations of tumor infiltrating lymphocytes **(C)** and IFN-γ production by tumor infiltrating T cells **(D)** were analyzed by flow cytometry. Data are expressed as Mean ± SD and represent 3–5 experiments with similar results. ns: no significant difference; *P < 0.05, **P < 0.01, ***P < 0.001 by One-way ANOVA. **(E,F)** CD4^+^CD25^+^FoxP3^+^ Tregs in tumors and spleens in mice bearing B16 **(E)** or J558 tumors **(F)**. B16 and J558 tumor establishment and treatment conditions were the same as described above **(A–D)**. Mononuclear cells from tumors and spleens were analyzed by flow cytometry. **P < 0.01 by One-way ANOVA.

To determine if intra-tumoral administration of AAV–IL-27 altered the tumor immune microenvironment, we examined the cellular components of tumor-infiltrating leukocytes in tumors from AAV–IL-27 or AAV-ctrl virus–treated mice using flow cytometry. As shown in Supplementary Figure S2A and Figure 3A, AAV–IL-27 intra-tumoral treatment significantly increased the percentage of CD45^+^ leukocytes and CD3^+^ T cells in B16 tumors. AAV–IL-27 treatment also enhanced tumor infiltration of CD3^–^NK1.1^+^ NK cells. We found that AAV–IL-27 treatment slightly decreased CD4^+^ T cells and significantly increased the infiltration of CD8^+^ T cells into B16 tumors. Moreover, we found that T cells, particularly CD8^+^ T cells in B16 tumors receiving AAV-IL-27 treatment produced more IFN-γ (Figure 3B). Similarly, we found that injection of AAV-IL-27 into established J558 tumors resulted in more CD45^+^ leukocyte infiltration. Among the CD45^+^ population, CD3^+^, CD4^+^, CD8^+^ T cells, and CD3^–^CD49b^+^ NK cells had increased (Supplementary Figure S2B and Figure 3C), and tumor infiltrating CD4^+^ and CD8^++^T cells produced more IFN-γ in AAV-IL-27 treated tumors (Supplementary Figure S3A and Figure 3D).

We previously showed that i.m. injection of AAV-IL-27 leads to systemic IL-27 production, which caused depletion of Tregs in peripheral lymphoid organs and tumors (Zhu et al., 2018). To determine if intra-tumoral injection of AAV-IL-27 depletes Tregs, we used flow cytometry to examine Tregs in spleens and tumors from treated mice. As shown in Figure 3E, intra-tumoral injection of AAV-IL-27 resulted in reduction of Tregs both in tumors and spleens. Similarly, we also found that AAV-IL-27 injection into established J558 tumors resulted in reduction of Tregs in both tumors and spleens (Supplementary Figure S3B and Figure 3F). Thus, intra-tumoral delivery of AAV–IL-27 induces systemic reduction of Tregs, and increases tumor infiltration of T and NK cells and enhances their effector functions.

### AAV-IL-27 Treatment Induces T Cell Expression of CXCR3

**FIGURE 4:**
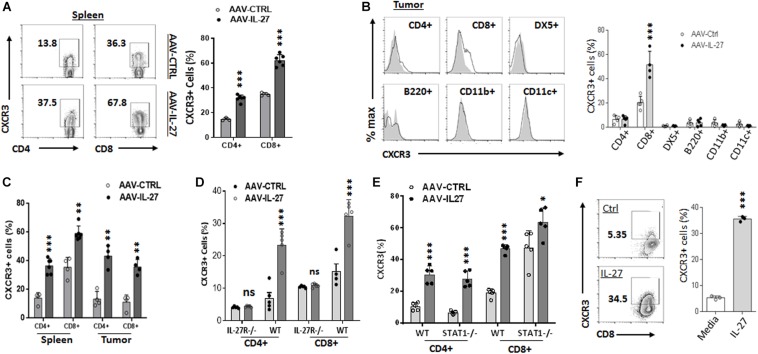
AAV–IL-27 therapy induces CXCR3 expression in T cells. **(A,B)** C57BL6 mice were injected with B16.F10 tumor cells (2 × 10^5^/mouse) s.c. 7 days later, mice were treated with AAV–IL-27 or AAV-ctrl virus intra-tumorally at a dose of 2 × 10^11^ DRP/mouse. Three weeks after AAV injection, mice were sacrificed and their spleens **(A)** and tumors **(B)** were analyzed for the expression of CXCR3 in T lymphocytes and other leukocytes by flow cytometry. **(C)** J558 cells (5 × 10^6^) were injected into BALB/c mice s.c. Five days later, mice were treated with AAV-IL-27 or AAV-ctrl virus intra-tumorally at a dose of 1 × 10^11^ DRP/mouse. Three weeks after viral injection, mice were sacrificed and their spleens and tumors were analyzed for the expression of CXCR3 in T lymphocytes by flow cytometry. **(D)** C57BL6 and IL-27R^–/–^ mice were injected with B16.F10 tumor cells (2 × 10^5^/mouse) s.c. Seven days later, mice were treated with AAV–IL-27 or AAV-ctrl virus intra-tumorally at a dose of 2 × 10^11^ DRP/mouse. 21 days after viral injection, mice were sacrificed and their spleens were analyzed for T cell expression of CXCR3 by flow cytometry. **(E)** AAV-IL-27 or AAV-ctrl virus (2 × 10^11^ DRP/mouse) was injected into Stat1^–/–^ or control BALB/c mice i.m. Three weeks after AAV injection, T cell expression of CXCR3 in spleens was analyzed by flow cytometry. **(F)** Spleen cells from P1CTL Tg mice were activated with P1A peptide (0.1 μg/ml) for 5 days *in vitro* in the presence or absence of IL-27 (50 ng/ml), and CXCR3 expression on P1CTL cells were analyzed by flow cytometry on day 5. Ns: no significant difference. ***P < 0.001 by One-way ANOVA.

Since AAV-IL-27 treated tumors had increased infiltration of T cells, we examined whether increased recruitment of T cells into tumors was a mechanism. CXCR3, a chemokine receptor for the interferon-inducible chemokines CXCL9, CXCL10, and CXCL11, is known to play key roles in T cell trafficking to tumors (Franciszkiewicz et al., 2012). We therefore examined if AAV-IL-27 intra-tumoral injection induced CXCR3 in T cells. As shown in Figure 4A, we detected significant levels of CXCR3 in CD4^+^and especially CD8^+^ T cells in spleens of AAV-ctrl treated mice. AAV-IL-27 treatment significantly increased the expression of CXCR3 in both spleen CD4^+^ and CD8^+^ T cells. In tumors, IL-27-induced CXCR3 expression was only found on T cells, especially CD8^+^ T cells, but not on other leukocytes (Figure 4B). Similarly, intra-tumoral injection of AAV-IL-27 into established J558 tumors resulted in induction of CXCR3 spleen and tumor T cells (Supplementary Figure S4 and Figure 4C). To determine if induction of CXCR3 is IL-27-specific, we treated WT and IL-27R^–/–^ mice with AAV-IL-27 or AAV-ctrl virus, and found that AAV-IL-27 only induced CXCR3 in CD4^+^ and CD8^+^ T cells in WT, but not IL-27R^–/–^ mice (Supplementary Figure S5A and Figure 4D). Interestingly, we found that AAV-IL-27-induced CXCR3 expression in T cells was independent of Stat1, because AAV-IL-27-treatment could induce CXCR3 expression in T cells from Stat1^–/–^ mice (Supplementary Figure S5B and Figure 4E). Finally, we found that co-culture of tumor-antigen P1A-specific P1CTL cells (Bai et al., 2003) with IL-27 *in vitro* resulted in significant upregulation of CXCR3 in P1CTL cells (Figure 4F), suggesting that IL-27 can directly induce CXCR3 in tumor-specific T cells.

### AAV–IL-27 Intra-Tumoral Delivery Overcomes Anti–PD-1 Resistance

**FIGURE 5:**
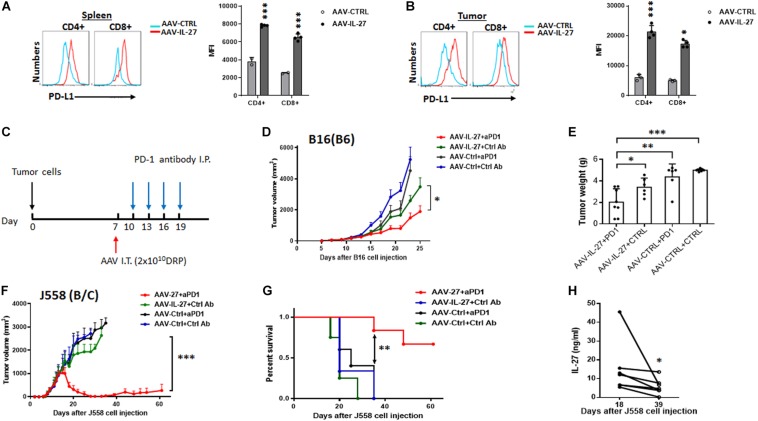
Intra-tumoral injection of AAV–IL-27 induces PD-L1 expression in T cells and enhances tumor sensitivity to anti-PD-1 therapy. **(A,B)** Flow cytometry analysis of CD4^+^ and CD8^+^ T cells in spleen **(A)** and tumors **(B)** from C57BL6 mice that were treated intra-tumorally with AAV-IL-27 or AAV-ctrl virus. Average mean fluorescence intensity (MFI) of PD-L1 expression in T cells from each group of mice was also shown. **(C)** Schematic diagram of intra-tumoral AAV-IL-27 and anti-PD-1 combination therapy. Mice are injected with tumor cells s.c. Seven days later, mice with established tumors are treated with AAV–IL-27 or AAV-ctrl virus intra-tumorally. Starting on day 10, mice are also treated with 250 μg/mouse of anti–PD-1 (RMP1-14) or an isotype-matched control antibody (2A3) i.p. at 3-day intervals for up to 4 times. **(D,E)** Synergy of anti-PD-1 and intra-tumoral AAV-IL-27 in inhibiting the growth of B16.F10 melanoma. AAV–IL-27 or AAV-ctrl virus intra-tumoral treatment were at a dose of 2 × 10^11^ DRP/mouse. Tumor volume changes **(D)** and mice tumor weight **(E)** were recorded. **(F,G)** Synergy of anti-PD-1 and intra-tumoral AAV-IL-27 in inhibiting the growth of J558 tumors. AAV-IL-27 or AAV-ctrl virus intra-tumoral treatments were at a dose of 5 × 10^10^ DRP/mouse. Tumor volume changes **(F)** and mice survival **(G)** were recorded. Data were expressed as Mean ± SD of at least five mice in each group. Data shown represent 2–3 experiments with similar results. Ns: no significant difference. *P < 0.05, **P < 0.01, ***P < 0.001 by One-way ANOVA or Log-rank test. **(H)** IL-27 concentration in peripheral blood from BALB/c mice receiving intra-tumoral AAV-IL-27 and anti-PD-1 combination therapy were examined.

We previously found that i.m. injection of AAV-IL-27 could induce PD-L1 expression in T cells, which unexpectedly overcome tumor resistance to anti-PD-1 therapy (Zhu et al., 2018). To test if intra-tumoral delivery of AAV-IL-27 induced PD-L1 in T cells, we analyzed PD-L1 expression in T cells in treated mice using flow cytometry. We found that CD4^+^ and CD8^+^ T cells in spleens and tumors of mice receiving AAV–IL-27 intra-tumoral treatment indeed upregulated PD-L1 expression (Figures 5A,B). To determine if IL-27–induced PD-L1 expression in T cells prevented the effectiveness of IL-27 local therapy, C57BL6 mice with established B16 tumors received the following treatments: AAV-ctrl virus + control mAb; AAV-ctrl virus + anti–PD-1; AAV–IL-27 virus + control mAb or AAV–IL-27 virus + anti–PD-1. Antibodies were injected into mice at a dose of 250 μg/mouse i.p. at 3-day intervals starting on day 10 (Figure 5C). As shown in Figure 5D, we found that B16 tumors grew progressively in mice treated with AAV-ctrl + ctrl Ab and AAV-ctrl + anti–PD-1; while in AAV–IL-27 + ctrl Ab–treated mice, slight inhibition of tumor growth was observed. AAV–IL-27 + anti–PD-1–treatment most significantly inhibited tumor growth, which also resulted in significantly reduced tumor weight at the end of the experiment (Figure 5E). We used a similar regimen to treat mice with established J558 tumors. A suboptimal dose of AAV (2 × 10^10^DRP) was used since high dose AAV-IL-27 could induced complete tumor rejection. As shown in Figure 5F, mice treated with AAV–IL-27 + control mAb showed reduced tumor growth compared with mice treated with AAV-ctrl virus + control mAb or AAV-ctrl virus + anti–PD-1. Most significant inhibition of tumor growth was observed in mice treated with AAV–IL-27 + anti–PD-1. Overall, AAV–IL-27/anti–PD-1 combination therapy resulted in tumor-free survival of 70% of mice (Figure 5G). We collected serum from some mice receiving combined treatment on days 18 and 39, during which time their tumors reached peak followed by tumor volume reduction. As shown in Figure 5H, significant reduction of IL-27 in blood was observed when the tumor volume decreased or disappeared. Thus, in the two tumor models that are resistant to anti–PD-1 therapy, we found that AAV-IL-27 local therapy and anti–PD-1 combination could induce complete tumor rejection or better tumor growth inhibition when a low dose of AAV-IL-27 was used.

### AAV-IL-27 Intra-Tumoral Administration Significantly Enhances Anti-tumor Efficacy of Tumor Infiltrating T Cells

**FIGURE 6:**
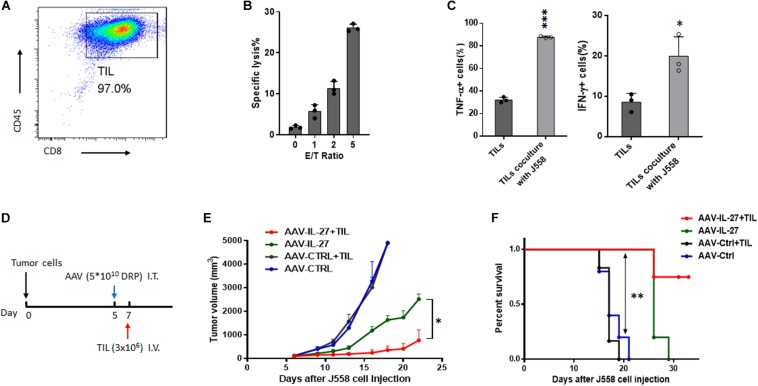
Intra-tumoral injection of AAV–IL-27 enhances tumor-infiltrating T lymphocyte therapy. **(A)** Flow cytometry analysis of tumor-infiltrating lymphomcyte (TILs) isolated and expanded from J558 tumors grown in BALB/C mice. **(B)** TIL effector functions were quantify by flow cytometry-based cytotoxicity assay. **(C)** TILs from J558 tumors produced IFN-γ and TNF-α upon co-culture with J558 tumor cells *in vitro*. **(D)** Schematic diagram of intra-tumoral AAV-IL-27 and adoptive transfer of TILs. Mice were injected with J558 tumor cells s.c. Five days later, tumor-bearing mice were treated with AAV–IL-27 or AAV-ctrl virus intra-tumorally followed by injection of TIL T cells i.v. **(E,F)** Tumor growth **(E)** and survival of mice **(F)** bearing J558 tumors treated with intra-tumoral AAV-IL-27 and TILs. Data shown represent three independent experiments with *n* = 5 mice per group. *P < 0.05, **P < 0.01 by One-way ANOVA or Log-rank test.

Adoptive transfer of tumor infiltrating T cells (TILs) is an effective therapy for cancer patients (Rosenberg and Restifo, 2015). Our previous research (Liu et al., 2013) suggests that IL-27 significantly enhances the survival of tumor antigen specific CD8^+^ T cells *in vivo*, which indicates that AAV-IL-27 intra-tumoral administration could also be used as an adjuvant for T cell adoptive transfer therapy for cancer. We first generated tumor infiltrating lymphocytes (TILs) from established J558 tumors by MACS beads-based purification of CD8^+^ T cells followed by *in vitro* expansion. As shown in Figure 6A, TILs generated this way were of >97% purity. The TIL CD8^+^ T cells showed strong cytotoxicity to J558 tumor cells (Figure 6B), and produced TNF-α and IFN-γ upon co-culture with J558 tumor cells (Figure 6C). To determine if AAV-IL-27 intra-tumoral delivery enhances T cell adoptive transfer therapy, we first injected J558 cells into BALB/c mice to establish tumors and treatments were carried out as outlined in Figure 6D. AAV-IL-27 or AAV-ctrl virus was injected into tumors on day 5 when the tumors were established. On day 7, TIL CD8^+^ T cells were injected i.v. into mice treated with AAV-IL-27 or AAV-ctrl. As shown in Figure 6E, mice treated with AAV–IL-27 + TIL CD8^+^ T cells showed most significant tumor growth inhibition compared with mice treated with AAV-ctrl virus + TIL cells or AAV-IL-27 or AAV-ctrl alone. Prolonged survival of mice (Figure 6F) were observed in AAV-IL-27/TIL CD8^+^ T cell combination–treated mice. Thus, AAV-IL-27 intra-tumoral delivery showed significant synergy with T cell adoptive transfer therapy.

## Discussion

A critical problem for systemic delivery of cytokines for cancer therapy is toxicity (Waldmann, 2018). For instance, IL-2 is the first effective immunotherapy of human cancer but it is also known to be very toxic (Rosenberg, 2014). IL-12 exhibits potent anti-tumor activity (Colombo and Trinchieri, 2002) via promoting Th1/Tc1 response (Colombo and Trinchieri, 2002; Del Vecchio et al., 2007) and enhancing T cell trafficking to tumors through induction of chemokines (Hu et al., 2018). However, systemically delivered IL-12 causes fatal toxicity (Car et al., 1995; Ryffel, 1997). To avoid this problem, researchers have designed various strategies (Kerkar et al., 2010; Zhang et al., 2015; Zhao et al., 2019) to target IL-12 to tumors. However, unless IL-12 is confined to tumor site, systemic toxicity appears to be unavoidable (Momin et al., 2019). As an IL-12 family of cytokine, IL-27 shares some similarities with IL-12. For instance, IL-27 also promotes Th1/Tc1 responses and induces T cell infiltration into tumors via a variety of mechanisms (Yoshimoto et al., 2015; Zhu et al., 2018). However, unlike IL-12, IL-27 is considered to be a cytokine with low toxicity (Yoshimoto et al., 2015). Indeed, we recently reported (Zhu et al., 2018) that systemic delivery of IL-27 using rAAV could inhibit tumor growth and significantly enhance cancer immunotherapy in a variety of mouse tumor models without causing significant toxicity. Although our study (Zhu et al., 2018) suggests that high concentrations of IL-27 in blood were very well tolerized by mice, a potential caveat of systemic delivery of IL-27 using rAAV is that there is no practical method to terminate IL-27 production when its biological activity is no longer needed. To avoid this problem, we tested direct injection of AAV-IL-27 into tumors in this study. We found that in mice receiving AAV-IL-27 intra-tumoral injection, IL-27 was produced in tumors and released to blood. However, in mice whose tumors were completely rejected, IL-27 serum levels were significantly reduced or diminished (Figures 2G, 5H). These results suggest that intra-tumoral injection of AAV-IL-27 could (1) achieve similar efficacy as AAV-IL-27 systemic administration and (2) avoid the unwanted problem of continued high level IL-27 production. Thus, intra-tumoral delivery of AAV-IL-27 appears to be a feasible approach for enhancing anti-tumor immunity.

In this study, we found that intra-tumoral injection of AAV-IL-27 enhanced NK cells and especially CD8^+^ T cell infiltration in tumors. Moreover, we found that intra-tumoral AAV-IL-27 injection caused reduction of Tregs both in peripheral lymphoid organs and tumors. In a previous study (Zhu et al., 2018), we reported that systemic delivery of AAV-IL-27 led to depletion of Tregs in peripheral lymphoid organs and tumors via down-regulation of IL-2 signaling. Since intra-tumoral injection of AAV-IL-27 results in IL-27 release to blood, we believe the same mechanism applies here. We also observed that in mice that rejected J558 tumors, protective immunity was established (Figure 2H). This result suggests that intra-tumoral injection of AAV-IL-27 induces long term anti-tumor immunity in protected mice. We believe a number of mechanisms, including Treg reduction-induced additional T cell priming and IL-27 induced T memory stem cells (Liu et al., 2017), are responsible for induction of T cell memory.

Increased expression of CXCR3 in peripheral T cells suggests that increased T cell trafficking of T cells into tumors is the major mechanism for enhanced T cell infiltration into tumors after AAV-IL-27 intra-tumoral injection. In this case, CXCR3^+^ tumor-specific T cells accumulate into tumors through interaction with CXCR3 ligands (CXCL9, CXCL10, and CXCL11) (Franciszkiewicz et al., 2012). Previously, IL-27 was shown to induce CXCR3 in Tregs (Hall et al., 2012). In this study, we show that IL-27 directly induces CXCR3 expression in conventional T cells including tumor antigen specific CD8^+^ T cells (Figure 4). Since IL-27 activates the Stat1-T-bet axis (Hibbert et al., 2003; Kamiya et al., 2004), and T-bet directly transactivates CXCR3 (Beima et al., 2006; Lewis et al., 2007), we postulated that IL-27 directly induces T cell expression of CXCR3 via IL-27R-Stat1-T-bet axis. Unexpectedly, we found that in the absence of Stat1, IL-27 could still induce CXCR3 in both CD4^+^ and CD8^+^ T cells (Figure 4E). These results suggest that IL-27 can also signal through transcription factors other than Stat1 in T cells to induce CXCR3. Regardless of the mechanisms, our study suggests that intra-tumoral injection of AAV-IL-27 enhances CXCR3-mediated T cell infiltration and directly enhances T cell effector functions.

IL-27 is known to induce T cell expression of PD-L1 (Hirahara et al., 2012), and PD-L1-PD-1 interaction among T cells may inhibit T cell effector functions thereby limiting IL-27-mediated anti-tumor efficacy. However, our recent study (Zhu et al., 2018) suggests that this same activity renders tumors more susceptible to anti-PD-1 therapy. In this work, we found that (1) intra-tumoral delivery of AAV-IL-27 also induced PD-L1 in T cells in peripheral lymphoid organs and tumors, and (2) AAV-IL-27 and anti-PD-1 showed synergy in inhibiting tumor growth (Figure 5). Thus, the limitation of IL-27-induced PD-L1 expression in T cells can serve as an opportunity for developing novel combination therapy.

Adoptive transfer of TIL T cells is a well-established therapy for patients with solid tumors such as melanoma (Rosenberg and Restifo, 2015). In a standard protocol, a pretreatment of recipients with chemotherapy drugs is usually needed to deplete Tregs and make room for T cell homeostatic proliferation (Dudley et al., 2005). Consistent with this notion, we found that adoptive transfer of TILs alone was insufficient to induce tumor regression in non-lymphopenic mice (Figure 6E). However, intra-tumoral injection of AAV-IL-27 showed significant synergy with TIL therapy in the absence of pretreatment of recipient mice. This outcome is due to a number of mechanisms. First, as described above, IL-27-induced CXCR3 upregulation can enhance T cell trafficking into tumors. Second, IL-27 can directly stimulate TIL T cells, enhancing their survival ability and IFN-γ production, as we previously demonstrated (Liu et al., 2013; Li et al., 2015). Third, IL-27-mediated depletion of Tregs can bypass the need of lymphodepletion prior to T cell transfer. Nevertheless, our results suggest that intra-tumoral injection of AAV-IL-27 in combination with TIL adoptive transfer is a potential combination for cancer therapy.

Taken together, we evaluated if directly injecting AAV-IL-27 into tumors could lead to inhibition of tumor growth. In the two tumor models (B16.F10 and J558) tested, intra-tumoral delivery of high dose AAV-IL-27 showed potent anti-tumor activity in mice bearing plasmacytoma J558 tumors and modest anti-tumor activity in mice bearing B16.F10 tumors. Importantly, in mice whose tumors were completely rejected, IL-27 serum levels were significantly reduced or diminished, and protective immunity was established. Moreover, we found that intra-tumoral injection of AAV-IL-27 showed significant synergy with anti-PD-1 antibody or T cell adoptive transfer therapy in inhibiting tumor growth. Thus, intra-tumoral delivery of AAV-IL-27 is a feasible approach for enhancing anti-tumor immunity and can be used alone and in combination with anti-PD-1 antibody or T cell adoptive transfer for the treatment of cancer.

## Data Availability Statement

All datasets generated for this study are included in the article/Supplementary Material.

## Ethics Statement

The animal study was reviewed and approved by Ohio State University Animal Care and Use Committee.

## Author Contributions

AH and MD performed most of the experiments. JZ helped and performed flow cytometry analysis. J-QL produced rAAV and helped with mouse work. XP helped with statistical analysis. KG helped with experimental design. X-FB generated funding support, designed experiments, analyzed data and wrote the manuscript.

## Conflict of Interest

The authors declare that the research was conducted in the absence of any commercial or financial relationships that could be construed as a potential conflict of interest.

## Supplementary Material

FIGURE S1 Immunofluorescence analysis of GFP expression from tumors receiving AAV-GFP. Mice with established B16 tumors were treated with AAV-GFP (5^∗^10^9^DRP), 7 days later, the injection sites of tumor tissues were examined for the expression of GFP under a confocal microscope. Upper panel: tumor tissue from an untreated tumor; lower panel: tumor tissue from an AAV-GFP treated tumor.

FIGURE S2 Representative flow cytometry data showing analysis of tumor infiltrating leukocytes. **(A)** B16.F10 cells (2 × 10^5^) were injected into C57BL/6 mice s.c. Seven days later, mice were treated with AAV-IL-27 or AAV-ctrl virus intra-tumorally at a dose of 2 × 10^11^ DRP/mouse. Mice were sacrificed on day 21, and populations of tumor infiltrating leukocytes were analyzed by flow cytometry. **(B)** J558 cells (5 × 10^6^) were injected into BALB/c mice s.c. Seven days later, mice were treated with AAV-IL-27 or AAV-ctrl virus intra-tumorally at a dose of 5 × 10^10^ DRP/mouse, and mice were sacrificed on day 21, and populations of tumor infiltrating leukocytes were analyzed by flow cytometry. Data shown are representative of 3–5 experiments.

FIGURE S3 Representative flow cytometry data showing analysis of IFN-γ producing T cells **(A)** and Tregs **(B)** in mice bearing J558 tumors. Data are representative of three experiments with similar results.

FIGURE S4 Flow cytometry analysis of CXCR3 expression in T cells from mice bearing J558 tumors. Data are representative of three experiments with similar results.

FIGURE S5 Flow cytometry analysis of CXCR3 expression in T cells from IL-27R^–/–^ and Stat1^–/–^ mice. **(A)** C57BL6 and IL-27R^–/–^ mice were injected with B16.F10 tumor cells (2 × 10^5^/mouse) s.c. 7 days later, mice were treated with AAV–IL-27 or AAV-ctrl virus intra-tumorally at a dose of 2 × 10^11^ DRP/mouse. 21 days after viral injection, mice were sacrificed and their spleens were analyzed for T cell expression of CXCR3 by flow cytometry. **(B)** AAV-IL-27 or AAV-ctrl virus was injected into Stat1^–/–^ or control BALB/c mice i.m. Three weeks after AAV injection, T cell expression of CXCR3 in spleens was analyzed by flow cytometry. Data are representative of two experiments with similar results.
