# Supplementary material for: Intra-Tumoral Delivery of IL-27 Using Adeno-Associated Virus Stimulates Anti-tumor Immunity and Enhances the Efficacy of Immunotherapy
Source: Front Cell Dev Biol. 2020 Mar 27;8:210. doi: 10.3389/fcell.2020.00210 (PMC7118910; doi:10.3389/fcell.2020.00210)
Supplement: FIGURE S1 — Immunofluorescence analysis of GFP expression from tumors receiving AAV-GFP. Mice with established B16 tumors were treated with AAV-GFP (5∗109DRP), 7 days later, the injection sites of tumor tissues were examined for the expression of GFP under a confocal microscope. Upper panel: tumor tissue from an untreated tumor; lower panel: tumor tissue from an AAV-GFP treated tumor. [file Presentation_1.PPTX]

## Slide 1
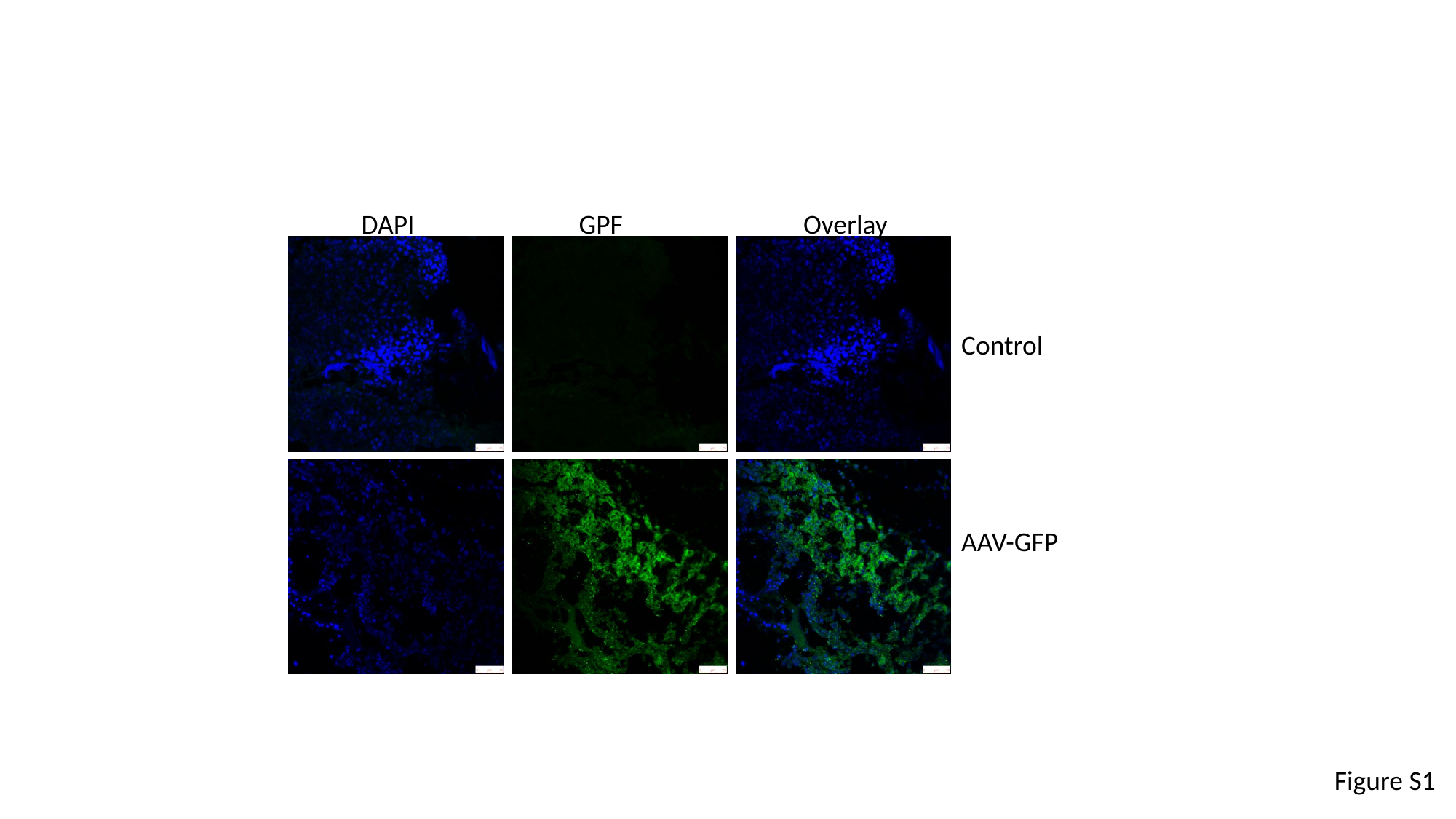

DAPI
GPF
Overlay
Control
AAV-GFP
Figure S1

## Slide 2
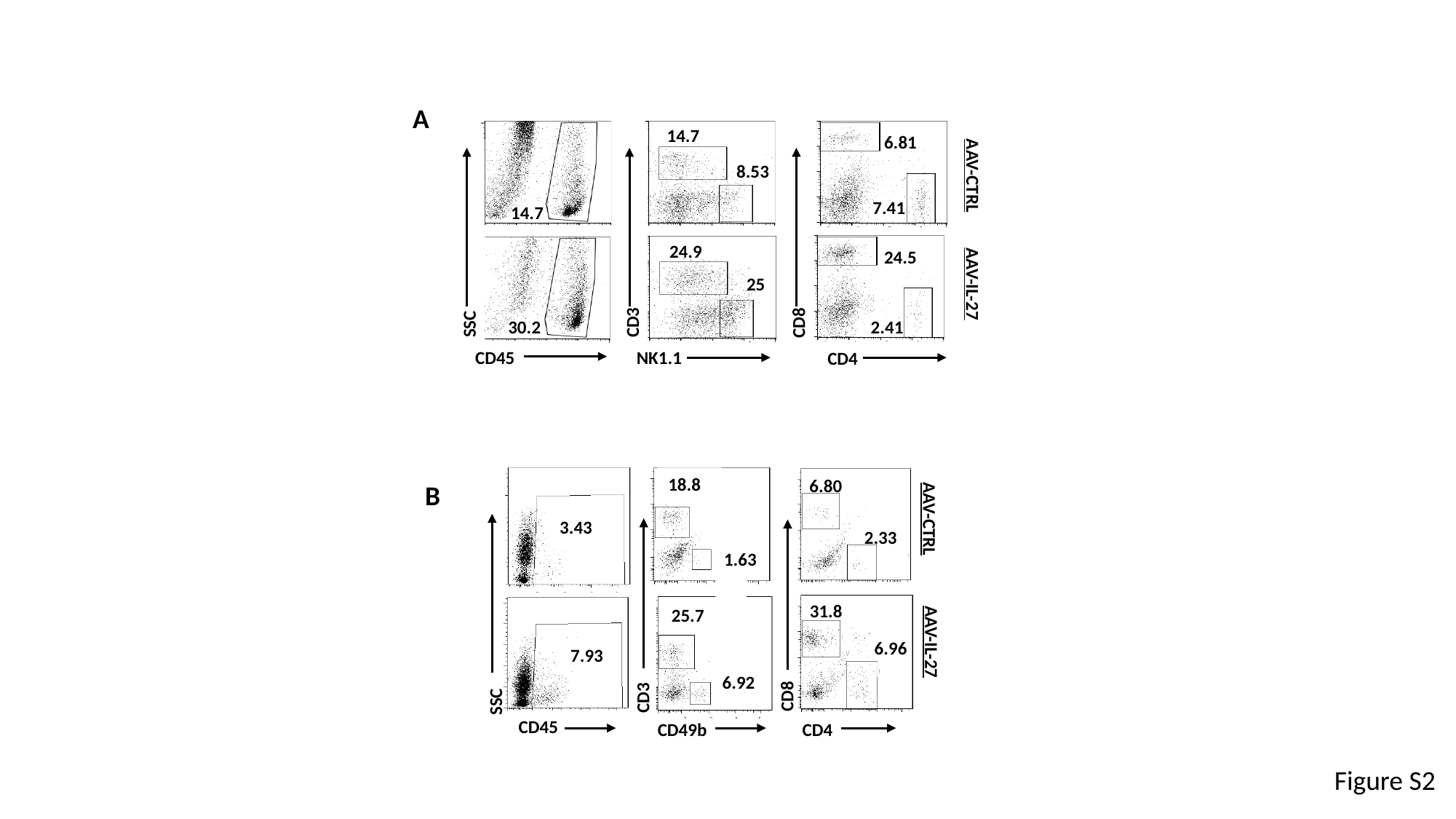

A
14.7
14.7
8.53
6.81
7.41
CD3
AAV-CTRL
AAV-IL-27
SSC
CD8
24.5
2.41
24.9
25
30.2
CD45
NK1.1
CD4
CD8
CD4
CD3
CD49b
AAV-CTRL
AAV-IL-27
SSC
CD45
18.8
6.80
B
3.43
2.33
1.63
31.8
6.96
25.7
7.93
6.92
Figure S2

## Slide 3
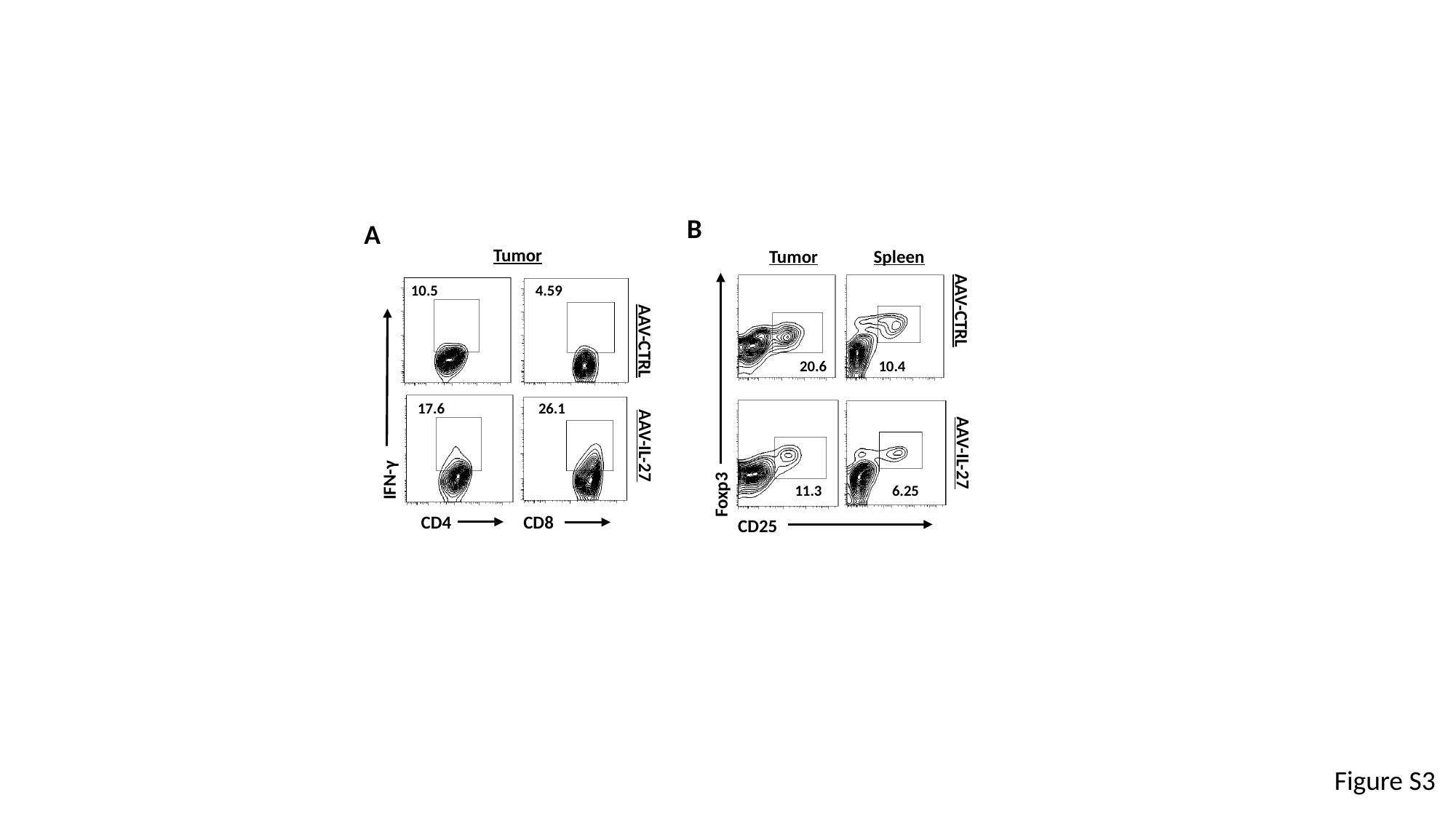

B
A
Tumor
IFN-γ
CD4
CD8
AAV-CTRL
AAV-IL-27
Spleen
Tumor
Foxp3
AAV-CTRL
AAV-IL-27
20.6
10.4
11.3
6.25
CD25
10.5
4.59
17.6
26.1
Figure S3

## Slide 4
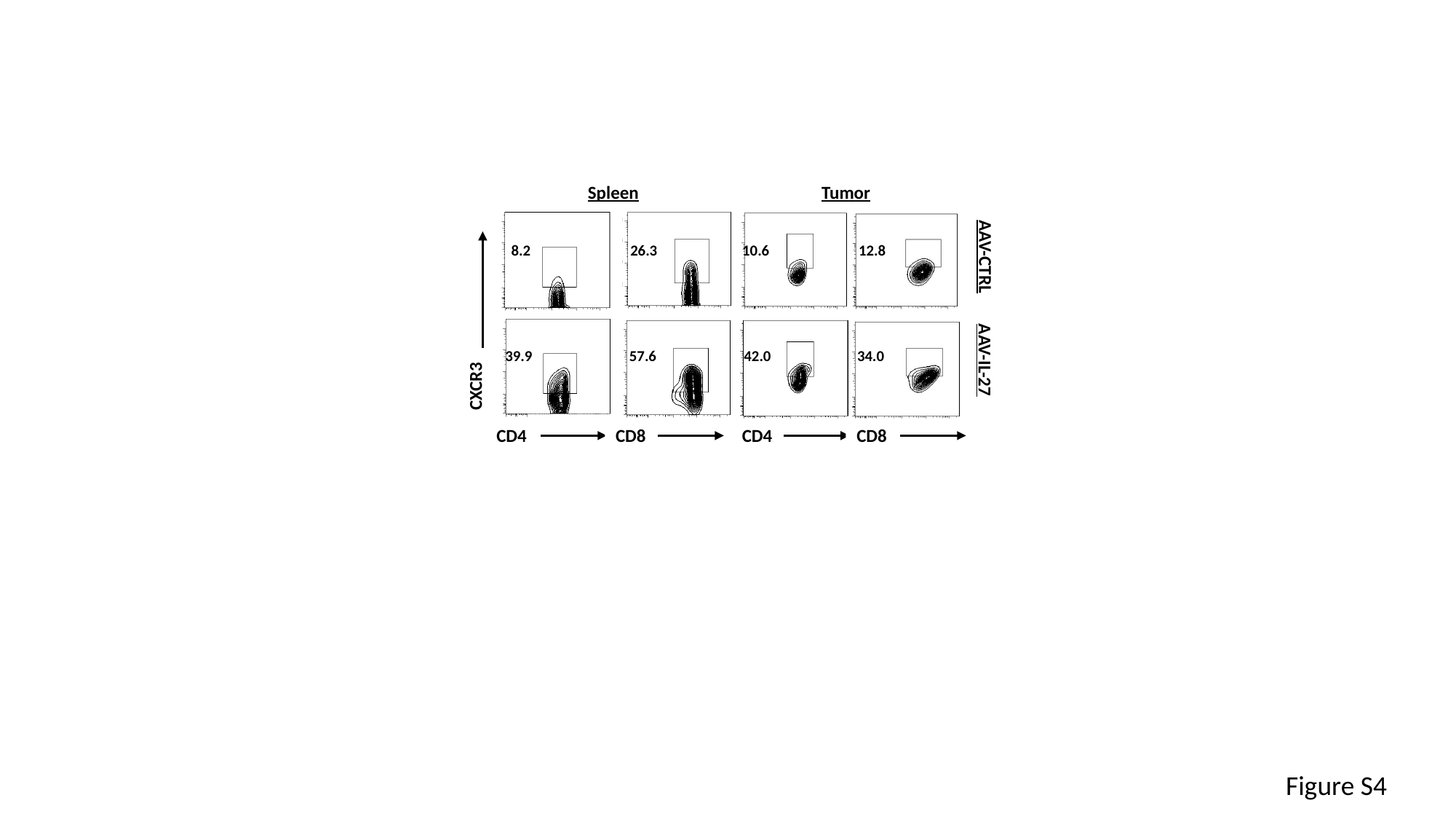

Spleen
Tumor
CXCR3
AAV-CTRL
AAV-IL-27
8.2
26.3
10.6
12.8
39.9
57.6
42.0
34.0
CD4
CD8
CD4
CD8
Figure S4

## Slide 5
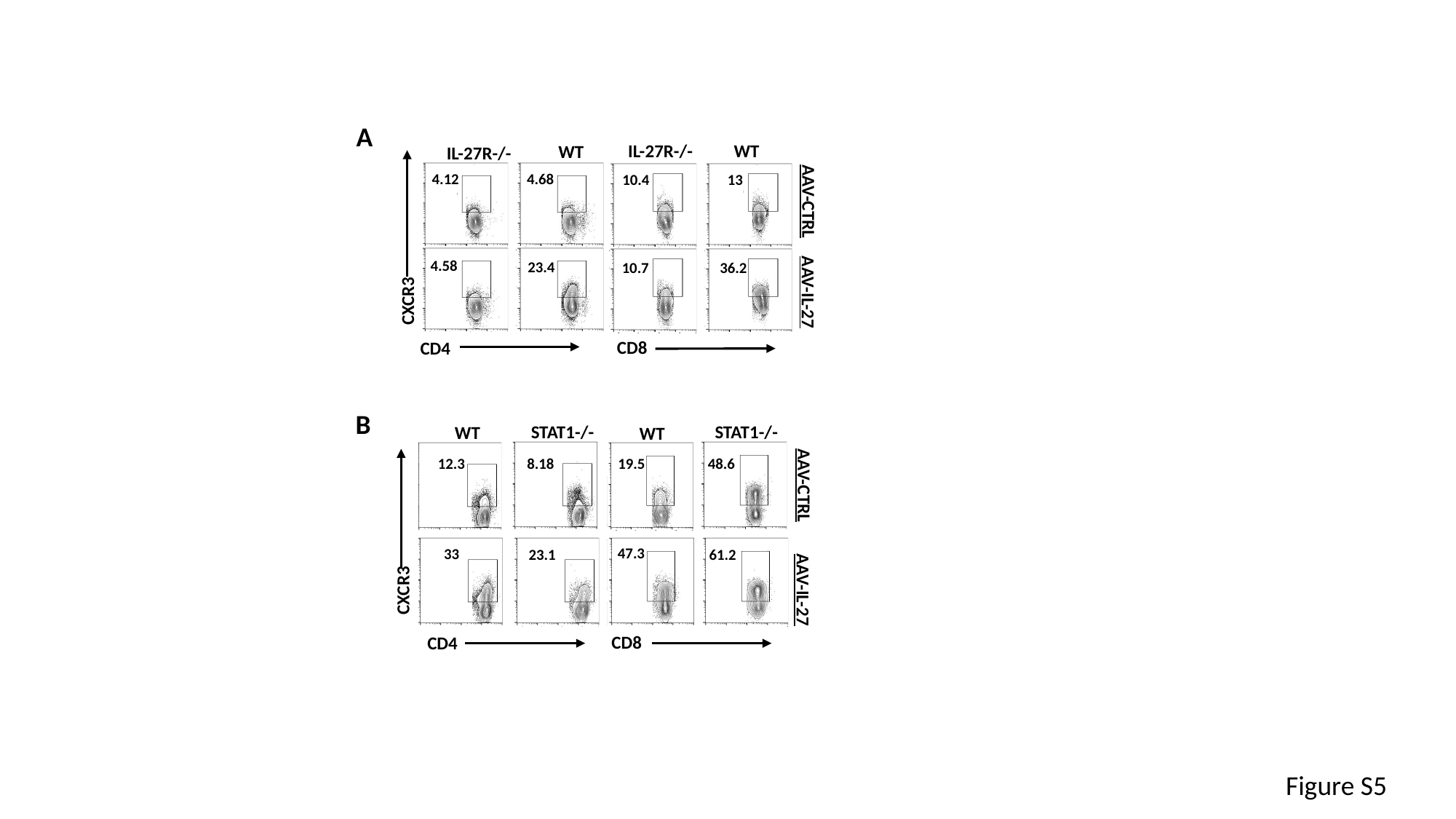

A
WT
IL-27R-/-
WT
IL-27R-/-
AAV-CTRL
AAV-IL-27
4.12
4.68
4.58
23.4
CD4
10.4
13
10.7
36.2
CD8
CXCR3
B
STAT1-/-
STAT1-/-
WT
WT
AAV-CTRL
AAV-IL-27
12.3
8.18
19.5
48.6
CXCR3
47.3
33
23.1
61.2
CD8
CD4
Figure S5
